# Genome Editing for Mucopolysaccharidoses

**DOI:** 10.3390/ijms21020500

**Published:** 2020-01-13

**Authors:** Edina Poletto, Guilherme Baldo, Natalia Gomez-Ospina

**Affiliations:** 1 Gene Therapy Center, Hospital de Clinicas de Porto Alegre, Porto Alegre 90035-007, Brazil; edinapoletto@gmail.com (E.P.); gbaldo@hcpa.edu.br (G.B.); 2 Post-Graduate Program in Genetics and Molecular Biology, Universidade Federal do Rio Grande do Sul, Porto Alegre 91501-970, Brazil; 3 Department of Pediatrics, Stanford University, Stanford, CA 94305, USA

**Keywords:** genome editing, gene therapy, mucopolysaccharidoses, lysosomal storage disease, CRISPR/Cas9, Hurler, Hunter, Zinc Finger Nucleases, viral vectors, non-viral vectors, hematopoietic stem cell transplantation

## Abstract

Genome editing holds the promise of one-off and potentially curative therapies for many patients with genetic diseases. This is especially true for patients affected by mucopolysaccharidoses as the disease pathophysiology is amenable to correction using multiple approaches. Ex vivo and in vivo genome editing platforms have been tested primarily on MSPI and MPSII, with in vivo approaches having reached clinical testing in both diseases. Though we still await proof of efficacy in humans, the therapeutic tools established for these two diseases should pave the way for other mucopolysaccharidoses. Herein, we review the current preclinical and clinical development studies, using genome editing as a therapeutic approach for these diseases. The development of new genome editing platforms and the variety of genetic modifications possible with each tool provide potential applications of genome editing for mucopolysaccharidoses, which vastly exceed the potential of current approaches. We expect that in a not-so-distant future, more genome editing-based strategies will be established, and individual diseases will be treated through multiple approaches.

## 1. Introduction: Therapeutic Principles in Mucopolysaccharidoses

The mucopolysaccharidoses (MPSs) are a group of genetic disorders caused by deficiencies in lysosomal enzymes, whose function is to degrade glycosaminoglycans (GAGs). Depending on the missing enzyme, the degradation of specific GAG species is blocked, generating a distinctive constellation of clinical symptoms, characteristic of each MPS form. Most MPSs are inherited as autosomal recessive disorders, except MPSII, which is X-linked [1].

Most MPSs, except MPSIIIC [2], result from deficiencies in soluble lysosomal enzymes that are secreted continuously into the extracellular space and blood, where they are taken up by adjacent cells and tissues. Consequently, cells with restored enzymatic capacity can “cross-correct” cells with the deficiency, a property known as cross-correction that forms the basis for most established and experimental therapies for MPSs [3]. This process of cross-correction explains why enzyme, when delivered systemically (such as in enzyme replacement therapy, or “ERT”) can improve symptomatology in some organs. Furthermore, cross-correction predicts that endogenous enzyme depots that could persistently produce enzymes could be effective at treating multiple affected tissues. Accordingly, cross-correction also explains why hematopoietic stem cell transplantation has been successful in some MPS diseases and why most viral and genome editing-based approaches aim to create such enzyme depots by targeting different organ systems [4].

An important property of MPSs is the relatively low therapeutic threshold, which is an essential consideration when developing gene therapy/gene editing-based therapeutic approaches for these disorders. Phenotypic characterization from healthy individuals with partial enzymatic activity and patients with mild phenotypes inform the target levels of enzymatic correction necessary for symptomatic relief. Though specific for every MPSs, restoration of enzyme level to approximately 10% of normal can be sufficient in most MPSs to produce clear benefit [5].

This ability to cross-correct the phenotype by targeting specific cell populations and organs, along with the low therapeutic threshold, has spurred development of a variety of therapeutic approaches for MPSs, and support the idea that in vivo and ex vivo gene editing approaches can be effective in this group of diseases. Genome editing, unlike ERT, has the promise of providing a one-time, definitive therapy for various genetic diseases. Compared to non-integrating viral therapy such as adeno-associated viruses (AAV), genome editing ensures that modifications are cemented into the genome, without risk of dilution with organ growth. In addition, ex vivo genome editing could circumvent some of the potential immunological complications of AAV. Finally, compared to randomly integrating viruses, e.g., lentiviruses, genome editing provides more specific (and therefore, more predictable) genetic modifications, and maintains the ability to preserve endogenous regulation of the corrected gene if desired.

## 2. Gene Editing: The basics

In this section, we present a brief description of the currently available genome editing platforms with a focus on those being developed for MPS. The discovery, evolution, and more technical aspects of each platform are not discussed here, as these have been extensively reviewed. A more in-depth discussion of each tool and its potential uses in MPS is provided elsewhere [6].

### 2.1. Genome Editing Platforms

**Figure 1:**
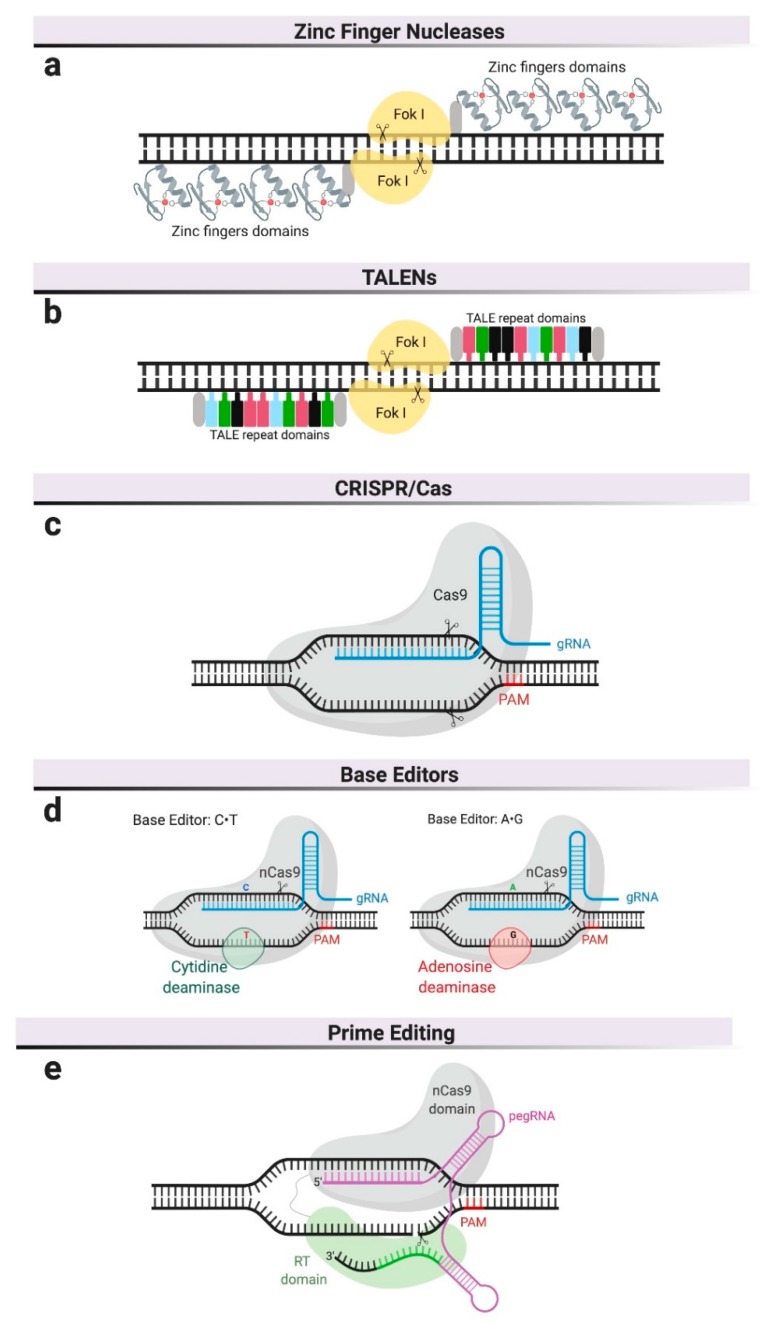
Genome editing platforms. (**a**) Zinc finger nucleases—zinc finger domains are fused to the restriction endonuclease, FokI, and require dimerization. (**b**) Transcription activator-like effector nucleases (TALENs) also use FokI as endonuclease but their DNA-binding domain is composed of repeats derived from transcription activator-like effectors (TALEs). (**c**) Clustered, regularly interspaced, short palindromic repeat (CRISPR)/Cas9 system uses a guide RNA (gRNA) to recognize specific DNA sequences and a Cas9 nuclease to cleave both DNA strands. DNA cleavage only occurs if the gRNA is adjacent to a Protospacer Adjacent Motif (PAM). (**d**) Base editors use inactive Cas9 or Cas9 nickase (nCas9), complexed with base-modifying enzymes and a gRNA. (**e**) Prime editors use a Cas9 nickase fused to reverse transcriptase and are complexed with pegRNA, which serves as gRNA and as template for reverse transcription.

Different genome editing platforms have been developed in the last decade [7]. The most relevant for therapeutic purposes are based on programmable nucleases and include the clustered regularly interspaced short palindromic repeat (CRISPR)–Cas9 system, zinc finger nucleases (ZFNs), transcription activator-like effector nucleases (TALENs), and the more recently described CRISPR-Cas9-based editors and prime editing (Figure 1). To date, only ZFNs and CRISPR–Cas9 have been used to develop therapies in MPSs beyond proof-of-concept studies and only ZFNs have reached the clinical stages of testing in human patients.

At the core of these nuclease-based editing technologies is the ability to create double-strand DNA breaks (DSB) at specific DNA sequences or genomic locations. Different platforms vary primarily in the mechanism by which the target DNA sequence is recognized (Figure 1). Engineered nucleases like ZFNs are customizable endonucleases consisting of an engineerable and sequence-specific DNA-binding domain, and the nuclease domain of a restriction enzyme, FokI [8] (Figure 1a). ZFNs require dimerization to create DSBs, resulting in laborious and expensive protein engineering for each potential target site, which has consequently prevented its widespread adoption in basic research and limited its development to a single commercial entity.

The field of genome editing made a significant advance in the earlies 2010s with the description of the bacterial Clustered Regularly Interspaced Short Palindromic Repeats (CRISPR)/CRISPR-associated system (Cas), in which a nuclease (Cas9) is guided to the target DNA sequence using RNA [9]. To achieve DSB at specific locations in eukaryotic genomes, the platform was engineered as a simple two-component system encoding an RNA element (called guide RNA or gRNA) and a Cas9 nuclease that has been codon-optimized for expression in eukaryotic cells [10,11,12,13]. The most widely utilized Cas9 in basic research and therapeutics are derived from *Streptococcus pyogenes* and *Staphylococcus aureus* [14]. DNA target recognition requires both complementarity to a 20 bp sequence in the gRNA and the presence of an adjacent short sequence (i.e., protospacer adjacent motif or PAM) in the DNA (Figure 1c). As a result of the RNA-based recognition, targeting different sequences only requires changes in the gRNA, a cheap and simple process that has driven the widespread adoption of this technology for basic research and therapeutic applications.

CRISPR-mediated base editing is a recent addition to the genome-editing toolkit. It does not rely on DSBs, even though it is based on the CRISPR/Cas9 system. This technology employs catalytically inactive Cas9 (not cut) or Cas9 nickase (cuts one of the two DNA strands) to target base-modifying enzymes, such as cytosine deaminase [15] or adenosine deaminase [16], to specific locations in the genome. Adenine and cytidine deaminases convert C∙G to T∙A base pairs, or vice versa, within a narrow window of the binding site (Figure 1d). This platform is, therefore, limited to pathogenic variants involving C or A residues in the vicinity of the PAM sequence required for Cas9 binding, so it is mutation-specific and not generalizable in diseases with many known causative mutations, such as MPSs. On the other hand, CRISPR-mediated base editing has the theoretical advantage of decreasing the probability of creating DSBs in unintended locations, commonly referred to as off-target sites.

The newest addition to the CRISPR tool kit is referred to as prime editing [17]. As with CRISPR-mediated base editing, prime editing does not rely on DSBs. Prime editors use a reverse transcriptase fused to a Cas9 nickase and a prime editing guide RNA (pegRNA) (Figure 1e). This pegRNA is a two-part RNA containing (a) a sequence complementary to the target site that directs Cas9 to its target sequence and (b) an additional sequence spelling the desired sequence changes. Once the RT-Cas9 protein is targeted to the genomic site and a nick in one of the DNA strands is created, the reverse transcriptase produces DNA complementary to the sequence in the pegRNA, which gets inserted at one of the cut ends and replaces the original DNA sequence. This technology has several advantages over the existing tools. Compared to the CRISPR-mediated base editing, prime editing can perform all transversion mutations (C→A, C→G, G→C, G→T, A→C, A→T, T→A, and T→G) as well as targeted deletions and insertions. Compared to tools that rely on DBSs, where NHEJ and HDR are competing repair processes resulting in varied outcomes, the editing outcomes are more precise and efficient, as they do not rely on exogenous donor DNA repair templates. In the absence of DSBs, this tool is potentially less genotoxic. Prime editing is predicted to correct up to 89% of known genetic variants associated with human diseases [17] though its specificity and potential for off-target modifications remains to be studied.

### 2.2. Multiple Genetic Modifications and Their Therapeutic Applications

**Figure 2:**
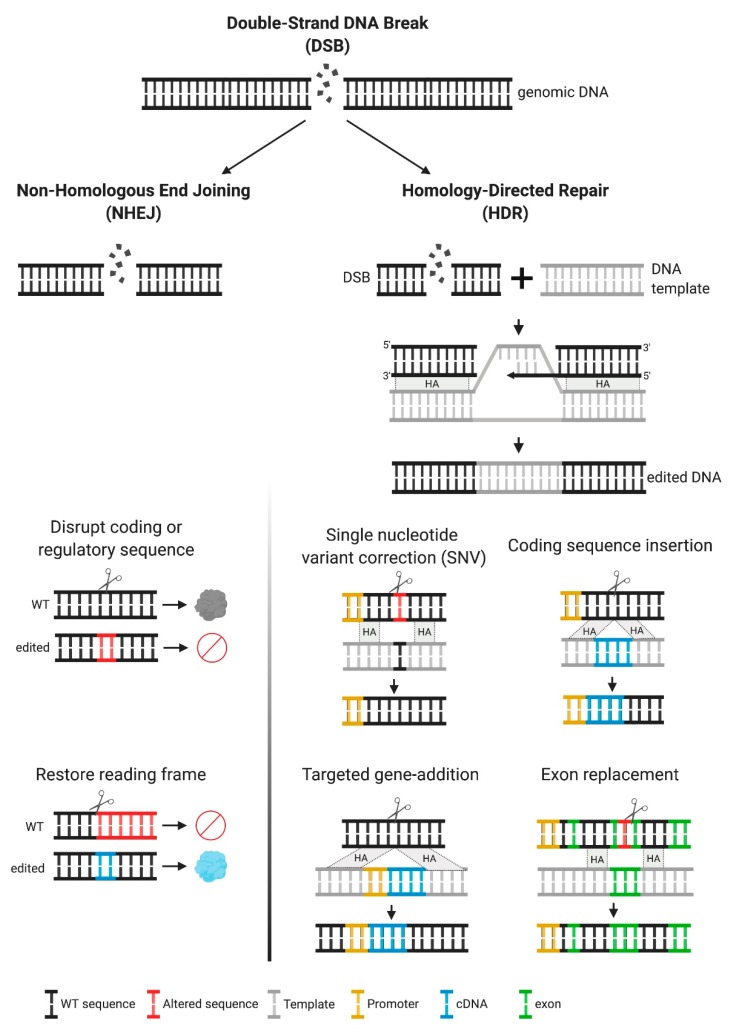
Multiple genetic modifications and their therapeutic applications. Upon a double-strand break (DSB), DNA can be repaired by two mechanisms, non-homologous end joining (NHEJ) or homology directed repair (HDR). The first mechanism frequently results in insertions or deletions (indels). Inducing indels can be used for disruption of coding sequences or to restore the reading frame, for frameshift mutations. For HDR to occur, a DNA template containing the desired modification, with homology arms flanking the target site is required. This approach is recommended when specific DNA modifications are intended, as this is an error-free repair mechanism. HDR can result in the following modifications, depending on the template used—single nucleotide variant (SNV), insertion of coding sequences under their own endogenous promoter control or alternative endogenous promoters, addition of genes (promoter + cDNA) in safe harbor loci, and replacement of partial coding sequences (one or multiple exons).

Once introduced into the cell, the ZNFs and Cas9/gRNA complexes translocate to the nucleus and cleave DNA at the intended sequences, generating a DSB, which triggers DSB-break repair mechanisms, primarily non-homologous end joining (NHEJ) or homologous recombination (HR) (Figure 2). NHEJ can result in imprecise repair, leading to small deletions or insertions (indels) at the break site (Figure 2). The therapeutic application of NHEJ-based genome editing is limited, particularly in diseases resulting from loss-of-function alleles and in which many pathogenic mutations have been reported, as in the MPSs disorders. Most commonly, NHEJ is used for the disruption of coding or regulatory sequences (Figure 2). Notably, this approach has reached clinical testing for hemoglobinopathies, such as sickle cell disease and beta-thalassemia, in which NHEJ-based genome editing is used to disrupt a regulatory sequence, to selectively turn off the expression of a repressor. This increases production of an alternative form of hemoglobin (fetal hemoglobin), which can ameliorate the phenotype [18]. In very specific circumstances, NHEJ can be used to create insertions or deletions of 1, 2, or 3 nucleotides that can restore the reading frame in a mutant gene (Figure 2). The efficacy of this approach depends on the frequency of the intended indel, compared to other potential indels and has been primarily aimed at targeting specific mutations in Duchenne muscular dystrophy [19,20], but not yet in MPSs.

In addition to NHEJ, repair of the DSB can be achieved by homology-directed repair (HDR). This type of repair is favored when the cell is supplied with an exogenous template containing the intended sequence changes and homology around the cut site (Figure 2). HDR allows for precise genetic changes with therapeutic potential, depending on the design of the exogenous homologous template. A common use of HDR is for single nucleotide variant (SNV) correction. This approach is particularly relevant in diseases with a common pathogenic mutation, and it is the most efficient HDR-based mechanism [21,22]. HDR can also be used to insert entire coding sequences under the control of endogenous promoters, thereby providing a single platform for all pathogenic variants. Coding sequences can also be integrated under alternate regulatory regions or under exogenous promoters, allowing for modifications to the temporal and spatial patterns of expression that might add therapeutic value [23]—an approach that is usually referred to as a “safe harbor” approach. Another therapeutic use of HDR is to perform partial cDNA insertions containing an abbreviated functional protein, or coding regions with mutational hot spots, a strategy that can be used in cases where the cDNA is long and full replacement is not feasible.

For most therapeutic applications, the desired outcome is for repair to be directed by a template DNA, resulting in precise edits. For genome editing that relies on DSBs (not CRISPR-mediated base editing or prime editing), outcomes of genome editing can have multiple byproducts, resulting in a mixture of NHEJ and HDR. In most cells, these processes are competing and NHEJ is generally the most efficient. This observation has been explained, at least in part, by the activation of DNA-damage responses such as the p53 activation resulting in cell cycle arrest or even apoptosis [24,25]. Much effort over the past few years has focused on shifting this balance from NHEJ to HDR [26,27,28,29,30,31,32,33,34]. Alternative strategies to achieve precise and efficient editing have relied on moving away from DSBs by using catalytically inactive Cas9 or Cas9 nickase, as in CRISPR-mediated base editing or prime editing. Both of these tools have a great therapeutic potential, but proof-of-concept studies of efficacy and safety are still lacking, particularly in MPS diseases.

### 2.3. Delivery Platforms: Ex Vivo vs. In Vivo Genome Editing

**Figure 3:**
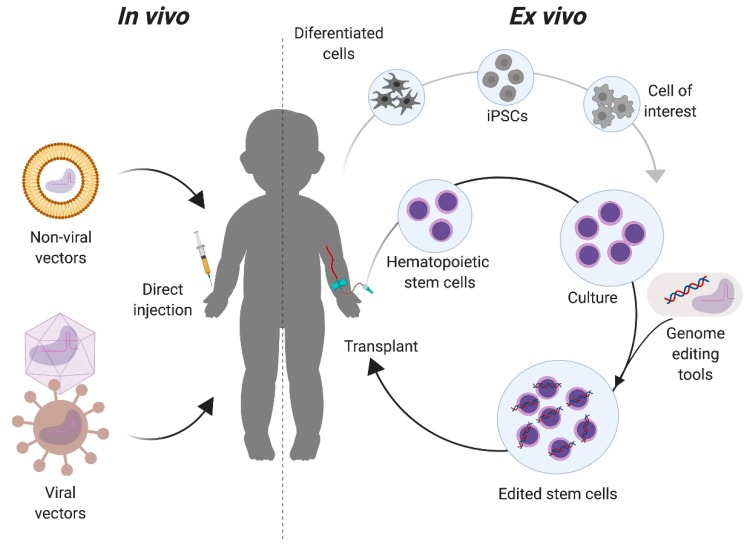
In vivo and ex vivo approaches for genome editing of Mucopolysaccharidoses. For in vivo strategies, the components required for the genome editing are complexed to delivery vectors that will be directly injected to the patient. These vectors can be non-viral, such as liposomes, or viral, such as adeno-associated virus. For ex vivo, cells with a regenerative potential, such as tissue stem cells (e.g., hematopoietic stem cells) or induced pluripotent stem cells (iPSCs) are collected from the patient and modified with the genome editing tools in vitro. Modified cells are then transplanted back to the patient for either autologous or allogeneic transplantation, depending on the cell source.

There are two primary approaches for targeting the genome editing components to the intended cells or tissues. In vivo approaches deliver the genome editing tools directly in the live organism, while in ex vivo delivery cells from the patient, suitable donor, or cell bank are modified outside of the body (Figure 3). With the in vivo approach, the correction of specific cells in the relevant organs depends highly on the tropism of the delivery vector used, the route of administration, and the physical as well as genomic accessibility of the target organ for genome editing. In the ex vivo approach, target cells with a regenerative potential must be isolated or be available for transplantation. Upon transplantation, these corrected cells could replace organs or migrate to the affected tissues (such as the brain). The choice of approach generally depends on the target organ. Advantages of the ex vivo approach include the control over which cells are targeted and the ability to fully characterize the editing outcomes, both intended (on-target) and unintended (off-target), in the targeted population. This approach has been extensively used in the hematopoietic system, where the isolation, culture, and transplantation of these cells is now routine [35,36]. However, not all organ functions can be replaced by transplantation of genome edited cells modified ex vivo. In these organs, e.g., the musculoskeletal and central nervous systems, an in vivo approach can be more effective.

## 3. Genome Editing vs. Other Therapeutic Approaches in MPS Disorders

### 3.1. Enzyme Replacement Therapy (ERT)

Early studies in cultured skin fibroblasts derived from MPS patients, serendipitously showed that a mixture of fibroblasts derived from patients with MPSI (Hurler syndrome) and MPSII (Hunter syndrome) had normal glycosaminoglycan metabolism, suggesting that cells with different lysosomal deficiencies could “cross-correct” each other [3]. Subsequent studies elucidated that lysosomal enzymes were secreted and could be internalized into cells via mannose-6-phosphate (M6P) receptors [3,37]. Based on these pivotal findings, recombinant lysosomal enzymes containing the M6P signal have been developed as therapies for MPSI [38], MPSII [39], MPSIVA [40], MPSVI [41,42], and MPSVII [43].

In MPS diseases, ERT has significant limitations in efficacy. Current formulations involve the intravenous administration of recombinant enzyme, most of which ends up in the reticuloendothelial cells of the liver and spleen, limiting its uptake in the affected tissues [44]. Specifically, intravenously-delivered ERT is mostly ineffective against central nervous system (CNS) manifestations (as it does not cross the blood–brain barrier) and in connective tissues such as cartilage and bone (due to poor vascularization). In addition, immune reactions to the recombinant enzyme and the development of neutralizing antibodies can decrease bioavailability [45]. Approaches to improve tissue targeting by engineering enzymes to cross the blood–brain barrier [46,47] and through alternative routes of administration (intrathecal or intracerebroventricular) [48] have been under development, in order to improve its efficacy, particularly in the CNS. Despite these improvements, ERT is an onerous therapy for patients as it requires life-long weekly or bi-weekly infusions, and is expensive for healthcare systems [49].

Unlike ERT, genome editing promises a one-time therapy for MPS and depending on the delivery platform (in vivo or ex vivo), it has the potential to treat organs such as the brain and bone. Similar to the ERT, challenges remain for efficiently targeting the genome editing components to the affected organs and for potential immune reactions to the expressed enzyme in the edited tissues, particularly in patients with null alleles [50].

### 3.2. Substrate Reduction Therapy

An alternative approach to ERT is substrate reduction therapy (SRT), a strategy that uses small molecule inhibitors to reduce the synthesis of stored metabolites [51].The most successful agents in this class are Miglustat and Eliglustat tartrate, both glucosylceramide synthase inhibitors that are effective at ameliorating disease manifestations in Gaucher disease type 1, a common lysosomal storage disease [52,53]. For MPS, the ability of SRT to provide therapeutic benefit remains unknown. Miglustat did not reduce ganglioside levels and failed to improve or stabilize behavior in a randomized trial in MPS III [54]. Non-specific inhibitors such as rhodamine B and genistein could reduce lysosomal storage in MPS mouse models [55,56], but failed to show any clinical effect [57]. Even if successful, SRT will need to be chronically administered, which is a disadvantage compared to one-time approaches such as gene therapy or genome editing. Compared to gene replacement strategies, it might be less likely to elicit immunological complications but side effects due to non-specific inhibition could be considerable [58].

### 3.3. In Vivo Gene Therapy with Adeno-Associated Viruses (AAV)

Adeno-associated viruses (AAVs) are viruses that commonly infect humans and appear to lack significant pathogenicity [59]. Gene therapy vectors using AAV can transduce dividing and non-dividing cells, and persist mostly in an extrachromosomal state without integrating into the genome, making them attractive vectors for gene delivery [60]. These vectors are typically delivered in vivo where the targeting to the affected organs is achieved by choosing serotypes with the appropriate tropism. In vivo gene therapy with AAV for MPS is currently being tested in clinical trials for MPSI (NCT03580083), MPSII (NCT03566043), MPSIIIA (NCT02053064), MPSIIIB (NCT03315182), and MPSVI (NCT03173521). As AAV rarely integrates into the genome, this approach is best at targeting organs that are not undergoing expansion through cell division such as the central nervous system or adult tissues where cell division is not expected to dilute the therapeutic gene. This is quite different from genome editing, where modifications are cemented into the genome, without risk of dilution with organ growth. Like all gene replacement strategies, AAV-mediated gene therapy and genome editing have the potential to elicit immune reactions to the delivery vectors or the transgenes.

### 3.4. Allogeneic Hematopoietic Stem Cell Transplantation

Allogeneic hematopoietic stem cell transplantation (allo-HSCT) replaces enzyme-deficient bone marrow cells with donor-derived enzyme-competent cells. Compared to ERT, allo-HSCT results in better outcomes by providing an endogenous source of enzyme in the vasculature, and by replacing the phagocytic cells of the monocyte macrophage lineage (osteoclasts, macrophages, and histiocytes) in the affected organs [61,62,63]. It has been most successfully used to treat severe MPSI (Hurler) where it prolongs survival, delays cognitive decline, and leads to improvements in hepatosplenomegaly, airway disease, and hearing loss [63,64,65]. However, allo-HSCT has not demonstrated significant benefit on musculoskeletal involvement, growth, or valvular disease [66]. Other caveats of allo-HSCT include finding compatible donors (which delays treatment) and the morbidity resulting from conditioning, graft versus host disease, and immunosuppression. Given the significant morbidity and mortality of the procedure, allo-HSCT has been limited to patients with severe MPSI, before age 2, and with normal cognitive function at the time of evaluation [67]. In addition to MPSI, allo-HSCT has resulted in positive clinical outcomes in patients with MPSIV, MPSVI, and MPSVII, but compared to other available therapies, the risks have outweighed the benefits [40,68,69]. Allo-HSCT was not found to prevent neurological decline in patients with severe MPSII, and MPSIII, where it might actually worsen symptoms [70,71].

### 3.5. Ex Vivo Lentiviral Modification of Hematopoietic Stem and Progenitor Cells

Given the potential benefit of allo-HSCT in some types of MPS, ex vivo modification of the patient’s own hematopoietic stem cells using integrating viruses or genome editing, to establish autologous transplantation, are being pursued. Such approaches have several advantages over ERT, SRT, and allo-HSCT—(1) achieving higher levels of enzyme expression, (2) eliminating the morbidity of graft rejection, graft-versus-host disease, and immunosuppression, and (3) earlier intervention.

To establish autologous HSCT in MPS, genetic modification of the patient’s cells is needed to correct the biochemical defect and restore enzyme activity. One way to accomplish this is to use integrating viruses [72]. Proof of the therapeutic potential of this strategy for MPS has been accomplished with lentiviral vectors. Specifically, for MPSI, preclinical studies in mouse models demonstrated improved efficacy, compared to transplantation using unmodified cells expressing endogenous levels of the enzyme [73,74]. This strategy is currently being tested in patients with severe MPSI (NCT03488394) and preliminary data from lentiviral delivery in HSPCs in humans, suggesting that this approach is effective in the CNS [75].

Delivery of lysosomal enzyme using lentiviruses constitutes an “untargeted” gene addition approach. Integrations into the genome are random or semi-random, raising concerns about the potential for tumorigenesis [76]. Furthermore, because of the variability in the location and number of integration sites in different cells, untargeted gene addition can result in heterogenous expression of the enzyme. As genome editing allows for precise, more targeted genetic modifications, it can result in more predictable transgene expression and theoretically less chance of insertional mutagenesis. Unlike gene addition using lentiviruses, genome editing provides the opportunity to modify the enzyme locus to preserve endogenous regulation of the corrected gene if desired, or to combine gene addition with precise knockout of other genes, to enhance efficacy.

## 4. From Proof of Concept Studies in Animal Models to Clinical Trials

For a complete summary of all preclinical and clinical studies using genome editing for MPSs, see Table 1.

### 4.1. In Vivo Approaches

The first studies describing in vivo genome editing for MPSs used the ZFN platform. An initial study reported a general approach for in vivo protein production, using ZFNs [23]. In this approach, the authors tried to circumvent the low efficiency of in vivo genome editing by integrating into a locus with high transcriptional activity. For this, they designed ZFNs targeting the albumin locus, using it as a safe harbor site. The genome-editing components were delivered in vivo via intravenous injection of adeno-associated virus 8 (AAV8). Using this method, they were able to produce supraphysiologic levels of several proteins, including alfa-galactosidase A (the enzyme deficient in Fabry disease), factor IX (hemophilia), and alpha-L-iduronidase (or IDUA, the enzyme deficient in MPSI).

Studies were then conducted in the MPSII and MPSI mouse models by using the same approach for the targeted insertion of the respective enzymes, Iduronate-2-sulfatase (IDS) and IDUA, into the albumin locus. For MPSII, three dose levels of recombinant AAV2/8 were tested, ranging from 2.5 × 10^11^ to 1.2 × 10^12^ viral genomes per mouse. Plasma IDS activity increased in a dose-dependent manner, with supraphysiological levels being observed in the higher-dose group (up to 200-fold normal). The enzyme produced was captured by other tissues (including the spleen, the heart, and the lungs) and successfully normalized the GAG levels. At a higher dose, an increase in brain IDS activity was observed, but it was not enough to reduce the GAG levels in this organ, despite improvements in behavioral parameters [77]. In MPSI mice, a dose of 1.5 × 10^11^ vector genomes (vg) of AAV2/8 with the ZFN and 2 × 10^12^ vg of an AAV2/8 with the hIDUA construct were administered intravenously in young mice (from 4 to 10 weeks of age). As observed in the MPSII mice, serum IDUA levels were increased to 9-fold normal and remained steady for up to 4 months. Tissue enzyme activity was significantly increased, and the GAG levels were normalized in all analyzed tissues, except for the brain, despite behavioral improvements [78].

These promising results in animal models suggested that this approach could be effective in humans, and the strategy is being tested in two clinical trials in MPSI (ClinicalTrials.gov, NCT02702115) and MPSII (NCT03041324). It is important to note that both ZFN products are intended only for patients with mild forms of the diseases who have little or no CNS involvement, as the enzymes produced from the liver are not expected to cross the blood–brain barrier. CHAMPIONS, the first trial to attempt in vivo genome editing in humans, is an ongoing Phase 1/2 clinical trial to determine if dose escalation of ZFNs is safe and tolerable in patients with MPSII. The trials aim to test four different cohorts with ascending doses of the genome editing components (ZFN1, ZFN2, and IDS donor). Interim analysis of the clinical data for the first three cohorts showed no serious adverse effects related to the drug, and other adverse events were mild or moderate and were eventually resolved. An RT-qPCR assay was able to detect the integration in the mid-dose cohort, but a genomic-based test failed. No measurable increases in plasma IDS were detected (except in one patient with transaminitis). Patients were maintained on their ERT therapy during the initial studies. Additional clinical data is being collected after withdrawal of ERT. However, 1 subject in cohort 2 was planning to restart ERT after approximately 3 months, due to fatigue and concurrent increase in GAGs [79,80]. EMPOWERS is an ongoing phase 1/2 clinical trial of ZFNs for MPSI (NCT02702115). It aims to test three different cohorts with ascending doses of the genome editing components (ZFN1, ZFN2, and IDUA donor). Interim analysis of the first three subjects was reported [81]. The preliminary results suggest that the treatment is safe although its efficacy remains to be proven. Plasma IDUA activity was measured but has not significantly changed from pre-treatment values. Data collection is ongoing.

CRISPR-Cas9 has also been used for preclinical studies in mice for in vivo genome editing in MPSI. The correction strategy was based on preliminary studies in human MPSI fibroblasts, where an HDR-based SNV correction approach was implanted using a liposome as vector, plasmids encoded the Cas9 nuclease and the gRNA, and a donor repair template aimed to correct one of the most common mutations found in MPSI patients with severe disease (p.Trp402Ter) [82,83]. To demonstrate efficacy in an MPSI mouse model, an HDR-based safe harbor approach was used by inserting the IDUA cDNA into the ROSA26 locus. Mice were treated with a single injection of liposome-complexed plasmids at 2–3 days of age. IDUA production was increased, compared to the untreated controls, with serum IDUA levels reaching 5–7% of wild-type mice for up to 6 months [84]. IDUA tissue levels were increased in all analyzed organs, except for the brain, followed by a similar pattern of reduction in the GAG levels. Despite low serum levels, the treated MPSI mice showed normalization of the cardiorespiratory function, which is the leading cause of death in patients. Some organs, such as the bones and the aorta, had partial improvements, with reduction in femur thickness and in elastin breaks. Organs traditionally known to be hard to correct, such as the heart valves and the brain, showed no improvement after therapy [85]. An additional study used CRISPR-Cas9 in vivo to target the common p.Trp402Ter mutation in a compound heterozygous mouse model of MPSI [86]. The strategy was shown to be partly efficacious in post-mitotic tissues like the heart.

### 4.2. Ex Vivo Approaches

**Figure 4:**
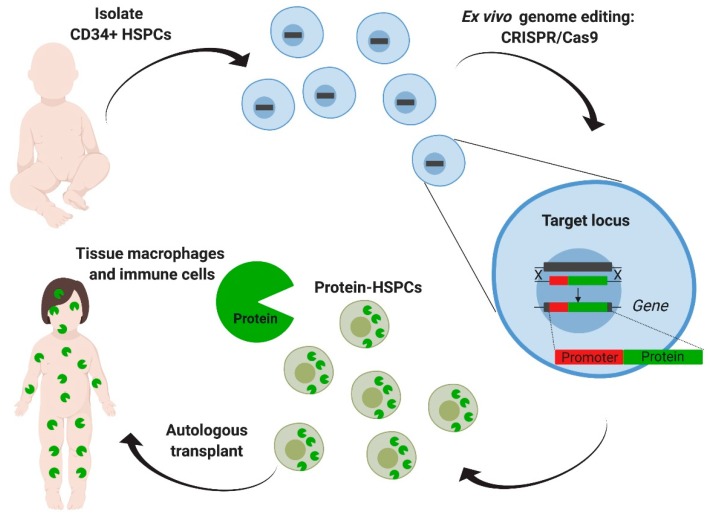
Ex vivo genome editing of hematopoietic stem cells targeting a safe harbor locus. Hematopoietic stem and progenitors cells (HSPCs) derived from the patient are targeted with CRISPR/Cas9 aiming the addition of the deficient enzyme’s coding sequence, driven by an exogenous constitutive promoter in the *CCR5* safe harbor locus. After transplantation, modified HSPCs will eventually engraft, reconstitute the hematopoietic system, and generate tissue macrophages and other immune cells that can produce and deliver the enzyme. This strategy eliminates the need for donor matching and decreases the risk of graft-versus-host disease. Furthermore, a safe harbor extends its applicability to other lysosomal diseases.

Ideal candidate cells for ex vivo modification are tissue-specific stem cells. Among these, hematopoietic stem and progenitor cells have been heavily studied as clinicians and researchers have extensive experience with their isolation, ex vivo manipulation, and transplantation [35,36]. Furthermore, hematopoietic stem cell transplantation (HSCT) in the allogenic setting has been shown to be a feasible enzyme reservoir in several metabolic disorders and MPSs [87,88,89]. Specifically, for MPSI, allogeneic HSCT has been shown to expand life expectancy and halt neurological decline. It is also the standard of care for patients under two years of age, who have a severe form of the disease and show a normal developmental quotient at the time of the evaluation [67]. Accordingly, the first genome editing ex vivo approach using hematopoietic stem cells was first established for MPSI. The goal of this approach is to establish autologous transplantation of genetically corrected cells by targeting the patient’s own hematopoietic stem cells and engineering them to produce high levels of the needed enzyme [90] (Figure 4). Compared to allogeneic HSCT, this overall strategy achieves higher levels of enzyme expression, eliminates the morbidity of graft-versus-host disease and immunosuppression, and can lead to earlier intervention by obviating the need for donor matching. Compared to ERT and the ZFN approaches described earlier, it provides enzymatic correction in the CNS.

In these studies, human hematopoietic stem and progenitor cells (HSPCs) were targeted using CRISPR-Cas9 to insert an IDUA expression cassette into the safe harbor locus, *CCR5* (Figure 4). The safe harbor allowed for expression of the enzyme outside the restrictions of the endogenous locus, as the goal was to engineer cells for supra-endogenous expression, which had been previously shown to enhance therapeutic potency [74]. A safe harbor also establishes a universal corrective approach for all patients with MPSI, as it circumvents the design for specific patient mutations. Finally, because the targeted locus does not change, the same genome-editing tools can be easily adapted to express other lysosomal enzymes, as there is no additional optimization of the gRNA and the donor repair template. To enhance the genome editing efficiency in human HSPCs, the researchers used gRNAs that were chemically modified with 2′-*O*-methyl 3′phosphorothioate [91] and *Streptococcus pyogenes* Cas9 protein complexed with the gRNA (RNP) and delivered into the cells by electroporation. The donor template for repair was delivered via adeno-associated virus 6 (AAV6) (Figure 4). When inserting an expression cassette where the IDUA expression was driven by the phosphoglycerate kinase (PGK) promoter, human HSPCs and the HSPCs-derived macrophages expressed 25-to-50-fold more IDUA than the unmodified cells. Serial transplantation studies showed that HSPCs modified in this manner retained the long-term repopulation and multilineage differentiation potential, confirming that this strategy can modify long-term stem cells and could constitute a one-time therapy for the disease. The efficacy of the edited HSPCs was examined in a model of MPSI capable of human cell engraftment. Transplantation of the edited cells led to a reconstitution of enzyme activity in serum, liver, spleen, and brain. GAG storage was also decreased in serum, liver, spleen, but not in brain. The transplanted mice demonstrated improvement in the bone pathology, neurobehavior (ambulation, short-term memory, and anxiety), and neuroinflammation. Together, this work provided specific evidence of safety and efficacy to support the optimization and development of this strategy into a clinical protocol to treat patients with MPSI and a platform approach to potentially treat other lysosomal storage disorders.

Other cell sources are beginning to be investigated. Induced pluripotent stem cells (iPSCs) are pluripotent stem cells generated directly from somatic tissues of patients and have the capacity to give rise to various cell types in the body (neurons, cardiomyocytes, immune cells, hepatocytes, skeletal muscle, etc.). These cells have been targeted in vitro in cellular models of MPSI, though efficacy studies are still lacking [92].

## 5. Challenges to the Clinical Adaptation of Genome Editing in MPSs

Many potential challenges still exist in the future application of therapeutic genome editing for MPSs. Within the preclinical stages of therapy development, appropriate assessment of efficacy and safety remains a challenge. Efficacy must be examined within the parameters of the intended therapeutic threshold in humans, as this might differ from current animal models, which is particularly important in MPSs, where murine models appear to have a lower threshold for biochemical correction and have a more permissible bone and CNS pathology. Not surprisingly, approaches that were shown to be effective in these models have failed to show results in humans. Challenges are specific to the delivery platform. For in vivo approaches, efficacy can be limited by the biodistribution of the available vectors and whether the vector biodistribution would be replicated in humans. Furthermore, genetic correction strategies that rely on HDR are less likely to work in post-mitotic tissues such as neurons. For ex vivo approaches, intrinsic challenges lie in the ability to find cells with a regenerative potential to target disease pathology. While this has been easy to conceive in the hematopoietic system, it has more challenging to achieve in the musculoskeletal and central nervous system.

Perhaps more importantly, there is a significant challenge in assessing the safety of therapeutic genome editing before translation to humans. Several aspects of safety that need to be evaluated, include specificity, tumorigenicity, and immunogenicity. A lingering concern of genome-editing technologies is their potential to create modifications at unintended genomic sites that could ultimately result in tumorigenicity. Many methods have been described to assess, in an unbiased manner the frequency of off-target modification and potential chromosomal abnormalities induced during the genome-editing process [94,95,96,97,98,99,100,101,102]. While studies have shown that the use of short-lived Cas9 (as protein) and mutant Cas9 with improved fidelity can significantly decrease and sometimes abrogate this off-target problem [90,103,104,105], there is still no gold-standard test or threshold. Ultimate assessment of tumorigenicity relies on pathological studies in animal models, but whether these studies accurately predict tumorigenicity is debated. Immunogenicity of the delivery vectors and Cas9 is another critical concern, particularly in the in vivo setting. Currently, most in vivo approaches rely on AAV for delivery of the genome editing tools. Specifically, AAV-neutralizing antibodies can reduce AAV-transduction, while CD8^+^ T cells directed to AAV capsid antigens can cause rejection of the AAV-transduced cells [59]. Similarly, preexisting antibodies and primed cytotoxic T cells have been found in healthy human donors to the *S. aureus* and *S. pyogenic* Cas9 [106,107]. For ex vivo approaches, immunological challenges lie in the indissoluble relationship between the origin of the cells and the organism. The therapeutic potential of edited human cells, the ultimate intended target, can only be faithfully assessed in the context of a human organism and immune system.

## 6. Conclusions and Future Directions

Gene editing holds the promise for precise, definitive, and sometimes curative therapies for patients affected with genetic diseases. This is especially true for patients affected by mucopolysaccharidoses where disease pathophysiology is highly amenable to correction. Preclinical studies have shown efficacy of in vivo and ex vivo approaches with different genome editing platforms. Though still not shown to be effective in humans, the fact that in vivo genome editing was first-in-humans for MPSs diseases is highly encouraging. While ex vivo modification of hematopoietic stem cells is still in preclinical stages, the strategy is based on 30 years of clinical and research experience in MPSI, supporting its potential use in treating CNS and skeletal symptoms in other MPSs disease. Autologous transplantation of genetically engineered cells will be safer than the current allogeneic option, and with additional engineering, its therapeutic potential could be enhanced so as to be a viable alternative for MPS patients who are not routinely considered to be transplantable. Challenges remain regarding the safety and efficacy of these tools for clinical translation. Many are not specific to genome editing, so the concurrent advancement of viral-based therapies will improve the therapeutic application of genome editing as well.

## Figures and Tables

**Table 1:** Genome editing studies for Mucopolysaccharidoses.

**Pre-Clinical Studies in Cell Models**										
**Disease**	**Affected Gene**	**Targeted Gene**	**Platform**	**Cell Type**		**Delivery Method**			**Genetic Modification**	**Reference**
MPS I	*IDUA*	*IDUA*	CRISPR/Cas9	Human fibroblasts		Plasmid-Liposome complex			SNV correction	[82]
MPS I	*IDUA*	*IDUA*	CRISPR/Cas9	Human fibroblasts		Plasmid-Liposome complex			SNV correction	[93]
MPS I	*IDUA*	*IDUA*	CRISPR/Cas9	mouse iPSCs		Plasmid-Liposome complex			Precise deletion	[92]
**Pre-Clinical Studies in Murine Models**										
**Disease**	**Affected Gene**	**Targeted Gene**	**Platform**	**In Vivo** ** Vs. Ex Vivo**	**Cargo and Vehicle**			**Genetic Modification**		**Reference**
MPS I	*IDUA*	*CCR5*	CRISPR/Cas9	ex vivo	RNP/AAV6			Gene addition		[90]
MPS I	*IDUA*	*ROSA26*	CRISPR/Cas9	in vivo	Liposome and plasmid vectors, IV			Gene addition		[84]
MPS I	*IDUA*	*IDUA*	CRISPR/Cas9	in vivo	2 AAV9 vectors, IV			SNV correction		[86]
MPS I	*IDUA*	*ALB*	ZFNs	in vivo	3 AAV2/8 vectors, IV			Gene addition		[78]
MPS II	*IDS*	*ALB*	ZFNs	in vivo	3 AAV2/8 vectors, IV			Gene addition		[77]
**Clinical Trials**										
**Disease**	**Affected Gene**	**Targeted Gene**	**Platform**	**In Vivo** ** vs. Ex Vivo**	**Cargo and Vehicle**	**Genetic Modification**	**Company**		**Trial Name**	**Clinicaltrials.gov Identifier**
MPS I	*IDUA*	*CCR5*	CRISPR/Cas9	ex vivo	RNP/AAV6	Gene addition	Stanford University		in the pipeline	
MPS I	*IDUA*	*ALB*	ZFNs	in vivo	3 AAV2/6 vectors, IV	Gene addition	Sangamo therapeutics		SB-318	NCT02702115
MPS II	*IDS*	*ALB*	ZFNs	in vivo	3 AAV2/6 vectors, IV	Gene addition	Sangamo therapeutics		SB-913	NCT3041324
IV: intravenous										
